# Decreased Adiponectin Levels in Early Pregnancy Are Associated with High Risk of Prematurity for African American Women

**DOI:** 10.3390/jcm11113213

**Published:** 2022-06-04

**Authors:** Yelizavet D. Lomakova, Xinhua Chen, T. Peter Stein, Robert A. Steer

**Affiliations:** 1 Department of Obstetrics/Gynecology, Rowan University School of Osteopathic Medicine, Stratford, NJ 08084, USA; lomako84@rowan.edu; 2 Department of Surgery, Rowan University School of Osteopathic Medicine, Stratford, NJ 08084, USA; tpstein@rowan.edu; 3 Department of Psychiatry, Rowan University School of Osteopathic Medicine, Stratford, NJ 08084, USA; steer@rowan.edu

**Keywords:** adiponectin, preterm delivery, ethnic difference, cytokine

## Abstract

The relationship of low maternal serum adiponectin levels with preterm delivery among a multi-ethnic group has not been extensively investigated. We examined ethnic differences in cytokine/adipokine profiles and whether they contribute to several adverse pregnancy outcomes, particularly preterm delivery. Data and samples were from a large prospective observational cohort (*n* = 1776) of young, generally healthy pregnant women (African American 36.4%, Hispanic 48.0%, Caucasian 15.6%). Serum cytokine/adipokine concentrations were measured at entry (mean gestational age of 16.83 weeks) using the Liminex xMap Technology. Multivariable analyses were performed. A significant difference in adiponectin level was observed among ethnic groups. African Americans had a decreased adiponectin and increased resistin levels compared to Hispanics and Caucasians (*p* < 0.05 to *p* < 0.0001 for each). Decreased adiponectin (lowest quartile) was positively associated with preterm delivery independent of usual risk factors (adjusted odds ratio (AOR) 1.46, 95% confidence interval (CI) 1.05, 2.04 for all preterm and AOR 1.84, 95% CI 1.07, 3.17 for early preterm births). The results were unchanged when women with preeclampsia were excluded. Similar results were observed in African Americans. Decreased adiponectin levels were not related to preterm birth in either Hispanics or Caucasians. Lower adiponectin levels were also significantly associated with an increased risk of developing gestational diabetes (AOR 1.72, 95% CI 1.05, 2.84) and preeclampsia (AOR 1.45, 95% CI 1.00, 2.14) in the whole cohort and in Caucasians. We did not find any consistent relationships between the other markers with outcome variables. Dysregulation in maternal adiponectin at early gestation is associated with an increased risk of preterm delivery. An ethnic difference in adiponectin levels may contribute to a higher preterm delivery rate in African American women.

## 1. Introduction

Preterm delivery and preeclampsia are serious pregnancy-associated problems and are recognized as leading contributors to perinatal morbidity and mortality [1,2]. Women who have experienced preterm delivery, preeclampsia, or both are also at an increased risk of cardiovascular disease (CVD) later in life [3,4].

Although the associations of inflammatory biomarkers with the risk of preterm delivery have been extensively examined in past decades, the evidence regarding which inflammatory biomarkers differ between preterm and term births is inconsistent and often based on small sample sizes of predominantly Caucasian women [5,6,7,8]. Many studies have shown that ethnic disparity in preterm delivery exists [1,9,10]. Whether ethnic differences in the cytokine-mediated inflammatory response contribute to ethnic differences in the risk of preterm delivery is not fully understood [9,10]. Results about the differences in the mean levels of inflammatory cytokines in cases of preterm compared to term births vary depending on the matrix used, i.e., serum or plasma as opposed to cytokines in other biological fluids, notably amniotic and cervicovaginal fluids [11,12,13,14].

Adiponectin is an adipokine with a strong anti-inflammatory property [15,16], and low adiponectin levels are associated with several adverse pregnancy complications, especially in gestational diabetes mellitus (GDM) [17,18,19]. Ethnic differences in maternal adiponectin levels have been found with normal pregnant women [20]. However, few studies have addressed the relationship between adiponectin and preterm delivery among pregnant women [21]. It is unknown whether the ethnic difference in adiponectin level contributes to the risk of preterm delivery in women with and without preeclampsia.

For this study, we selected several pro-inflammatory and anti-inflammatory markers that have shown mixed results in preterm delivery, or markers such as adiponectin and resistin that have not been widely examined in previous studies [5,6,7,8]. Since preterm delivery is a multifactorial syndrome, multiple markers have the potential to increase predictive power.

The purpose of this study was to investigate ethnic differences in cytokine profiles and how they contribute to adverse pregnancy outcomes, particularly preterm delivery. Specifically, we used data from a large cohort that included pregnant Hispanic, African American, and Caucasian women. Cytokines measured for this study included maternal serum IL6, IL8, IL10, TNFα, and Granulocyte-macrophage colony-stimulating factor (GMCSF), and the adipokines adiponectin and resistin. Also factored into the analysis were GDM and preeclampsia, both of which have a significantly increased risk for prematurity [2,22,23].

## 2. Materials and Methods

### 2.1. Study Design and Population

We conducted a prospective epidemiological study of healthy young pregnant women residing in one of the poorest cities in the continental United States [24]. The cohort of study participants enrolled between 1996 and 2006 was recruited from among patients enrolling at the Osborn Family Health Center, Our Lady of Lourdes Medical Center, and St John the Baptist prenatal clinic in Camden, NJ. The institutional review board at the University of Medicine and Dentistry of New Jersey (which later became Rowan University School of Osteopathic Medicine in 2013) approved the study protocol. Informed written consent was obtained from each participant at enrollment (on average at gestational week 13.48 ± 5.18, mean ± SD) after explanation of the nature and purpose of the study. The data and samples were stored in a repository since 2006, which allowed us to conduct the current analyses.

The criteria for inclusion were a positive pregnancy test, informed consent, and gestation at entry <20 weeks. A total of 3.5% of the women screened who had serious non-obstetric problems (e.g., lupus, type 1 or 2 diabetes, seizure disorders, malignancies, acute or chronic liver disease, drug or alcohol abuse, and psychiatric problems) were excluded. Eighty percent of the patients who were eligible agreed to participate. A total of 8.3% of participants dropped out after enrollment due to either a move from the area or an early pregnancy loss. A final total of 1776 participants with specimens available at entry to care were included in the present analyses.

### 2.2. Data and Blood Sample Collection

Participants were scheduled to see study research assistants before or after their regular prenatal visits at entry to care (14.2 ± 4.0 weeks, mean ± SD) and at gestational weeks 20 and 28. Data about socioeconomic, demographic, and lifestyle variables, such as education, occupation, marital status, income, cigarette smoking, alcohol consumption, dietary data, and reproductive history, were obtained by interviews at entry and updated at gestational weeks 20 and 28. Ethnicity was self-defined. Blood pressure was measured by clinic staff at each visit and abstracted from the medical records. Body mass index (BMI) (kg/m^2^) was computed based on self-reported pregravid weight; height was measured at entry to prenatal care. Maternal obesity was defined as BMI (kg/m^2^) ≥ 30.

The protocol for specimen collection has been previously described [25,26]. Briefly, fasting blood samples (>8 h) collected at entry to care (16.8 ± 6.4 weeks of gestation) were refrigerated and centrifuged at 4 °C. The serum and plasma samples were aliquoted and stored at −80 °C until assayed.

### 2.3. Analytic Assays of Cytokine and Adipokine

Cytokines (Il-6, IL-8, IL-10, TNF-α, and granulocyte-macrophage colony-stimulating factor (GMCSF)) and adipokines (adiponectin and resistin) were analyzed by two panels using the Luminex xMAP technology (Luminex Corporation, Austin, TX, USA) on the MagPix system.

Serum cytokine concentrations were determined by the Human High Sensitivity T-cell 5-plex magnetic bead panel (Category# HTSCMAG-28SK) and adipokines were determined by the human adipokine 2-plex magnetic bead panel (Category# HADK1MAG-61K) (EMD Millipore Corporation, Billerica, MA, USA). All analyses were performed according to the manufacturers’ protocols and in duplicate. Briefly, serum samples were thawed at 4 °C and centrifuged at 1000× *g* to remove any aggregate protein. The serum was diluted 1:2 in assay buffer and mixed beads were added to each well. A 7-point standard curve with serial dilutions was generated and quality control provided by the manufacturer were used to determine assay accuracy. The plate was incubated and agitated, then washed and re-incubated with detection antibodies. The plate was washed multiple times and the beads were re-suspended in the plate with 150 µL sheath fluid and analyzed using the Luminex Magpix.

According to the manufacturing protocol, there was no or negligible cross-reactivity between the antibodies for an analyte and any of the other analytes in each of the panels. The average intra coefficient variation (CV) was 5.5% (range 4.6–7.0%) and the average inter CV was 11.2% (range 8.2–13.2%) for cytokines. For adiponectin and resistin, the intra CV was 4.4% and 5.5%, and the inter CV was 10.1% and 11.3%, respectively. All analyses were performed according to the manufacturer’s protocols and in duplicate. The concentrations were calculated from best-fit standard curves generated from calibrators for each analyte in each assay by Milliplex analyst 5.1 software (VigeneTech Inc., Carlisle, MA, USA).

### 2.4. Definition of Pregnancy Outcomes

Pregnancy outcomes included in this study are described as follows. Preterm delivery is defined as delivery at <37 completed weeks of gestation based on the last menstrual period confirmed or modified by ultrasound evaluation [26]. Detailed information identifying women with spontaneous preterm delivery and medically indicated preterm delivery was obtained from the prenatal, labor, delivery, and newborn records including infant birth weight and sizes. Spontaneous preterm delivery was defined by the presence of intact membranes and regular contractions, and the absence of induction of labor or an elective caesarean section. Preterm premature rupture of membrane (pPROM) was defined as rupture of membranes before the onset of labor prior to 37 weeks’ gestation. Women with spontaneous labor and pPROM leading to preterm delivery were combined into the spontaneous preterm group. Information on reproductive history, including prior preterm delivery as well as the medical events during the current pregnancy, was also obtained by interview and/or abstracted from clinical records.

We classified the preterm birth as early (<34 weeks of gestation) and late (34–<37 weeks of gestation) preterm delivery to evaluate the influence of low adiponectin on varying gestational age.

Preeclampsia is identified by systolic blood pressure ≥140 mm Hg or diastolic blood pressure ≥90 mm Hg after 20 weeks’ gestation in a previously normotensive woman accompanied by new-onset proteinuria (>300 mg/24 h from a 24 h urine collection or ≥1+ protein by dipstick in two samples at least 4 h apart) [27].

The diagnosis of GDM was made by a two-step approach. Patients were initially screened by a 50 g oral glucose challenge test at 24–28 weeks of gestation. A 100 g oral glucose tolerance test (OGTT) was performed on the subset of women exceeding the glucose threshold value (>140 mg/dl at 1 h). The diagnostic criteria for GDM were based on the Carpenter/Coustan conversion, as recommended by the American Diabetes Association [28].

Participants with a term delivery and without a diagnosis of preeclampsia or GDM were defined as normal pregnancy controls.

### 2.5. Statistical Analysis

Parametric statistics were calculated for the continuous variables, and chi-square tests for independence were used for the categorical variables. Log10 transformations were performed for computing the means of the cytokines/adipokines when data were highly skewed. Analyses of variance (ANOVA) were used to assess the significance of linear trends and compare mean levels of each cytokine/adipokine marker among ethnic groups as well as comparisons between women with normal pregnancy outcomes and those who subsequently developed any of three adverse pregnancy outcomes.

Multiple logistic regression was used to estimate the odds ratio (OR) and 95% confidence interval (CI) for elevated cytokines or decreased levels of adiponectin with preterm delivery. We divided each of measured biomarkers into quartiles and used logistic regression to estimate the OR of adverse outcome in the highest or the lowest quartile using the other quartiles as the reference category. Separate models were performed to assess the association of biomarkers in preterm delivery women complicated with and without preeclampsia. We also conducted multiple polytomous logistic regression analyses to estimate the associations of elevated cytokine levels or decreased adiponectin with different types of preterm delivery, preeclampsia, or GDM as compared to normal pregnancy. This method models multiple levels of an outcome so that all adjusted odds ratios are estimated in the same model.

All multivariable analyses were also conducted controlling for potential confounding variables, which included maternal pre-pregnancy BMI, age, parity, cigarette smoking, and/or ethnicity where appropriate. The levels of significance were two-tailed and *p* < 0.05 was considered to be the lowest level of acceptable statistical significance. All of the statistical analyses were performed with SAS v.9.4 (SAS Institute, Inc., Cary, NC, USA).

## 3. Results

### 3.1. Maternal Characteristics and Birth Outcomes in Whole Cohort and by Ethnicity 

Maternal characteristics in the whole cohort and by ethnic group at entry are shown in Table 1. African American women were younger than Caucasians and Hispanics (*p* < 0.0001 vs. Caucasians and *p* < 0.01 vs. Hispanics) and had significantly higher pre-pregnancy BMIs and more obesity compared to Caucasian and Hispanic groups (*p* < 0.001 vs. Caucasians and *p* < 0.01 vs. Hispanics). There were small but significant differences among ethnic groups, including gestational age at entry (*p* < 0.05 African Americans vs. Caucasians) and systolic and diastolic blood pressure (*p* < 0.01 and *p* < 0.05 African Americans vs. Hispanics or Caucasians). There was a higher proportion of cigarette smoking and more private insurance in Caucasians (*p* < 0.0001 for each). No significant differences in gestational age at blood sampling, fasting blood glucose, and parity were found (*p* > 0.05).

The adverse pregnancy outcomes in women who subsequently developed GDM, preeclampsia, delivered a preterm infant, and infant birth weight by ethnicity are presented in Table 2. The mean infant birth weight was significantly lower in the African American group (*p* < 0.0001) vs. other groups. There was a higher proportion of African American women who delivered a preterm infant (all preterm <37 weeks) (13.00% vs. Hispanics (10.20%) and Caucasians (11.55%)), but the difference did not reach statistical significance. This was also observed for early preterm delivery (<34 weeks). Lastly, more African American women developed preeclampsia (*p* < 0.01), but fewer developed GDM (*p* < 0.001) as compared to other ethnic groups.

### 3.2. Ethnic Differences in Cytokine/Adipokine Concentrations

Because of positively skewed distributions in the measurements, log10 transformations were performed, and the final data presented in Table 3 have been reversed-logged for interpretive purposes. There were ethnic differences in adiponectin and resistin levels. African Americans had lower adiponectin and higher resistin and GMCSF vs. Hispanics or Caucasians (*p* for trend <0.05 to <0.0001 for each (Table 3)). Although African Americans had increased levels of IL8 (*p* < 0.05 vs. Caucasians) and TNF-α (*p* < 0.05 vs. Hispanics), the trends for all four cytokines were not statistically significant.

### 3.3. Cytokine and Adipokine Levels and Preterm Delivery

We compared the means of seven biomarkers measured with several outcome variables (Table S1). Adiponectin was the only marker that showed a significant difference among the outcomes. Women diagnosed with GDM had the lowest level of adiponectin compared to other outcome variables (*p* < 0.05 for each) as well as to the normal controls (*p* < 0.001). Adiponectin concentration was not significantly different between women with preterm delivery or preeclampsia and normal control (*p* > 0.05). The levels for the other six biomarkers were not statistically different comparing normal control and women with adverse outcomes.

Since the differences were only found with adiponectin concentration, more detailed analyses were subsequently performed to explore the associations of lower levels of adiponectin with preterm delivery. Separate models were used to evaluate the association between adiponectin and types of preterm (all, late, and early preterm) in all participants as well as by ethnic group (Table 4).

In all participants, decreased (lowest quartile, <11.34 µg/mL) adiponectin was associated with an increased risk of preterm delivery (adjusted odds ratio (AOR) was 1.46, 95% confidence interval (CI) was 1.05, 2.04) and early preterm birth (<34 weeks of gestation) (AOR 1.84, 95% CI 1.07, 3.17) after controlling for several potential confounding variables, including pre-pregnancy BMI, using term delivery as a reference. When the analysis was limited to preterm delivery patients without preeclampsia, the results remained significant (AOR 1.51, 95% CI 1.09, 2.09 for all preterm and AOR 1.82, 95% CI 1.03, 3.21 for early preterm). The same association was not observed in late preterm (34–<37 weeks of gestation).

When separate analysis was performed by ethnic group, we obtained similar results in African Americans. The significant association was only found with all preterm (AOR 1.80, 95% CI 1.10, 2.94) and late preterm births (AOR 1.84, 95% CI 1.02, 3.30). However, lower adiponectin was not significantly related to early preterm delivery (AOR 2.02, 95% CI 0.93, 5.17). In contrast, decreased adiponectin was not related to preterm birth in either Hispanics or Caucasians.

### 3.4. Cytokine/Adipokine Levels with Preeclampsia and GDM

We performed additional analyses using polytomous logistic regression to evaluate if cytokine/adipokine is associated with other adverse pregnancy outcomes, preeclampsia and GDM, after exclusion of women with preterm delivery. Women with lower adiponectin levels had a 55% increased risk of developing preeclampsia (AOR 1.55, 95% CI 1.06, 2.26) and greater than 2-fold increased risk for GDM (AOR 2.36, 95% CI 1.46, 3.79) when analyzed without adjustment for pre-pregnancy BMI. After pre-pregnancy BMI was controlled for, this finding on decreased adiponectin levels with a higher risk of developing preeclampsia (AOR 1.45, 95% CI 1.00, 2.14) and GDM (AOR 1.72, 95% CI 1.05, 2.84, *p* for trend <0.01) remained significant (Table 5).

There was also a significant negative relationship between IL10 and GDM. Increased IL-10 significantly reduced the risk of GDM by 2-fold (AOR 0.50, 95% CI 0.26, 0.96). There were no significant relationships in the levels of TNF-α, IL6, IL8, resistin, and GMCSF with preeclampsia or GDM (Table 5). Further analyses dividing participants by ethnic group only showed a negative relation of IL10 with GDM in Hispanic women (AOR 0.38, 95% CI 0.14, 0.99) and adiponectin with GDM in the Caucasian group (AOR 2.97, 95% CI 1.08, 8.17) (Table S2).

## 4. Discussion

In this prospective cohort of young and healthy pregnant women in a diverse ethnic population, our purpose was to examine the associations between multiple maternal circulating inflammatory biomarkers and preterm delivery and several other adverse pregnancy outcomes. We made three major observations: (1) decreased adiponectin concentration was associated with an increased risk of preterm delivery, particularly with a risk of early preterm birth; (2) the effect was significantly greater in African American women; (3) our findings are consistent with previous studies showing that a lower adiponectin level was associated with an increased risk of GDM [17,18,19]. We did not find any consistent relationships among any of the other biomarkers with outcome variables of interest.

### 4.1. Decreased Maternal Adiponectin Level Is Associated with Preterm Delivery 

Previous studies have shown that increased levels of several maternal cytokines, such as IL-6, IL-8, TNF-α, and CRP, as well as endothelial dysfunction markers, including soluble intercellular adhesion molecule-1 (sICAM-1), vascular cell adhesion molecule-1 (sVCAM-1), and soluble E-selectin (sE-selectin), were significantly associated with preterm delivery [5,6,7,26,29,30]. However, the findings are equivocal. A systematic review of 72 studies noted that none of the 30 biomarkers including inflammatory, placental proteins, hormonal, coagulation-related, and genetic biomarkers qualified as useful for clinical prediction in asymptomatic spontaneous preterm birth [6]. Menon et al. [8] analyzed data from 217 studies published over the past 40 years that included thousands of participants and 106 biomarkers for spontaneous preterm delivery (SPTD). The strongest finding was higher amniotic fluid concentrations of IL-6 in women with asymptomatic SPTD than women with term delivery [6,8,29]. Several studies have also suggested that biomarkers from cervicovaginal or amniotic fluid compared to maternal blood may prove to represent inflammation or infection events more accurately in the cervix and uterus than proteins in the maternal blood [11,12,13,14]. However, the test in amniotic fluid prior to labor requires invasive procedures that are not justifiable for asymptomatic women. Amniotic fluid collected at labor has limited prediction value [11,12]. In contrast, maternal blood, especially during early pregnancy, could have better prediction value and with a minimal risk to the mother and fetus [31,32].

Adiponectin as a biomarker for preterm delivery was not evaluated in any of the studies described in the above referenced meta-analyses [5,6,8,29]. A unique finding in our study was that women who had decreased adiponectin levels (lowest quartile) had 46% and 84% increased risk of having preterm and early preterm delivery after multivariable adjustment, respectively (Table 4). A similar result was found when patients without preeclampsia were analyzed (51% and 82%, Table 4).

In a cross-sectional study, Mazaki-Tovi et al. reported that a single episode of preterm labor that did not result in preterm delivery is associated with decreased maternal total adiponectin and high molecular weight adiponectin concentration (*p* < 0.01 to *p* < 0.001 vs. normal term delivery) [21]. The result persisted in patients with preterm labor regardless of the presence or absence of intra-amniotic infection/inflammation. Mazaki-Tovi suggested that low maternal adiponectin may be attributed to the process of preterm parturition instead of infection/inflammation alone. However, the maternal blood samples were collected at later second and third trimesters (25.5–31 weeks gestation) from that study [21]. We collected maternal samples at earlier gestation (~16 weeks, Table 1) in a large cohort with a multi-ethnic population.

Adiponectin is a circulating bioactive hormone secreted by adipocytes [15,16]. We cannot determine whether lower adiponectin is a cause or an effect in preterm delivery and preeclampsia due to the nature of our study. In animal and non-pregnant human studies, adiponectin prevented impaired endothelial function [33,34,35]. The endothelium is a major paracrine organ with critical roles in controlling vascular tone, inflammation, and smooth muscle cell proliferation [33]. Adiponectin inhibited angiogenesis and reduced oxidative stress in endothelium [33,34,35]. Since preterm delivery is a complex multifactorial syndrome [1,3], it is possible that impaired endothelial function is one of the mechanisms in preeclampsia or preterm delivery and may be indirectly regulated by adiponectin [26].

### 4.2. Ethnic Difference in Adiponectin and Risk of Preterm Delivery

There were marked ethnic differences in adiponectin levels (Table 3). African American women had lower adiponectin concentrations than other ethnic groups (*p* < 0.05). When we performed logistic regression analyses by ethnic group, a similar result was only obtained in African American women (AOR 1.80, 95%CI 1.10, 2.94 for all preterm and AOR 1.84, 95% CI 1.02, 3.30 for late preterm delivery, Table 4), but was not observed in Hispanic or Caucasian groups. These data suggest that ethnic disparity in the concentration of adiponectin may increase the risk of delivering preterm.

In an earlier prospective study, we reported that normal pregnant African American women had significantly decreased mean levels of adiponectin compared to Hispanics and/or Caucasians and a twofold increased chance of being in the lowest tertile compared to Caucasians [25]. Our data are also consistent with a study by Menon and colleagues [11]. They examined cytokines in amniotic fluid and found that racial differences existed in different type of cytokines. For example, IL-1beta and TNF-α concentrations were higher in cases of SPTD compared to term birth in African American women, but not in White women, suggesting a racial difference in the inflammatory response pathway may contribute to preterm delivery [11]. Similarly, Velez et al. [13] reported that patterns of cytokines profile from amniotic fluid differed between cases of preterm delivery and term controls in African American women, but reported no difference in White women [13]. The relative proportions of amniotic cytokines derived from maternal and fetal sources are unknown [11,12,13].

### 4.3. Decreased Adiponectin Is Associated with GDM and Preeclampsia

Women in the lowest quartile of adiponectin were at an increased risk of developing GDM (AOR 1.72, 95% CI 1.05, 2.84) (Table 5). It is well known that decreased maternal adiponectin is associated with GDM [17,18,19,20]. In addition, we observed that a higher IL10 level (highest quartile vs. other quartiles) was associated with a significantly decreased risk of GDM (AOR 0.50, 95% CI 0.26, 0.96). We did not find any significant association between other markers with GDM. When analyzed with low adiponectin by ethnicity, the results became non-significant (Table S2). This could be due to insufficient statistical power.

We also found that a decreased adiponectin level was associated with a higher risk of preeclampsia in the whole cohort without including the cases of preterm delivery (AOR 1.45, 95% CI 1.00, 2.14, Table 5). This finding was contrary to previous reports that found increased plasma adiponectin levels in women complicated with preeclampsia [36,37,38]. This might be because the populations, sample sizes, and gestational age of sampling differed from our study. Collectively, the findings suggest that adipokines may mediate the metabolic changes in women who developed preeclampsia.

### 4.4. Negative Findings in Other Cytokines with Preterm Birth and Limitations of Current Study

We did not find significant differences in other cytokine and resistin levels when comparing preterm birth to term delivery (Table S1). The balance of pro-inflammation and anti-inflammation during pregnancy is regulated and maintained by multiple factors [39]. Fetal sex, maternal nutrition, oxidative stress, and anti-oxidant status can influence maternal cytokine levels [40,41]. Our focus in the current study was the relationship between adverse pregnancy outcomes and cytokines. In addition, the placenta, the interface between the mother and fetus, is the source of hormonal regulators and the network of cytokines [39,42]. In in vitro experiments, a high level of expression of inflammation-related genes in adipose tissue and term placenta suggested that the placenta promotes the molecular interplay between maternal immune function and metabolism, which subsequently influences fetal growth [39]. Cytokines synthesized within the placenta can preferentially be released into the maternal circulation rather than the fetal circulation; most maternal cytokines are not readily transferred across the placenta in humans [39,42]. We were not able to identify the portion of the maternal cytokines that were from placenta. Thus, further studies will be needed to explore the other factors that influence cytokine levels and placenta function in regulating cytokines during pregnancy.

## 5. Conclusions

The present study is unique in examining multiple maternal inflammatory biomarkers during early gestation in a diverse ethnic population. Our findings suggest that dysregulation in maternal adiponectin is associated with an increased risk of preterm delivery. Furthermore, an ethnic difference in adiponectin levels may be a contributor to higher preterm delivery rates in African American women. Our findings underscore the need for future studies to determine how placental and fetal contribute to adiponectin levels in prematurity and the potential clinical usefulness of adiponectin as a biomarker for preterm delivery.

## Figures and Tables

**Table 1:** Characteristics of study participants according to ethnicity at entry to care.

Characteristics	All Participants	African American	Hispanic	Caucasian	* p * -Value
*n*	1776	646	853	277	
Age (years)	22.00 ± 5.23	21.33 ± 4.94 ^a,b^	22.11 ± 5.18 ^c^	23.31 ± 5.74	<0.0001
Pre-pregnant BMI (kg/m^2^)	25.72 ± 6.28	26.08 ± 7.06 ^c,b^	25.77 ± 6.79	24.68 ± 5.62	<0.01
Obese (BMI ≥ 30)	361 (20.33)	148 (22.11)	163 (19.11)	50 (18.05)	<0.01
Gestational age at entry (week)	13.42 ± 5.18	13.76 ± 5.08 ^d^	13.36 ± 5.34	12.84 ± 4.85	<0.05
Gestational age at blood sampling (week)	16.83 ± 5.47	16.92 ± 5.55 ^d^	16.99 ± 5.54 ^d^	16.12 ± 5.47	>0.05
Blood pressure (BP)					
Systolic BP	112.15 ± 11.42	113.80 ± 11.74 ^b^	110.82 ±11.00 ^d^	112.40 ± 11.40	<0.001
Diastolic BP	70.28 ± 8.65	71.31 ± 9.11 ^b,d^	69.67 ±8.17	69.78 ± 8.74	<0.001
Fasting blood glucose (mg/dL)	80.23 ± 16.57	80.19 ± 17.61 ^b^	79.88 ±14.87 ^d^	81.49 ± 18.99	>0.05
Cigarette smoking	335 (18.86)	124 (19.20)	105 (12.31)	106 (38.27)	<0.0001
Nulliparas	695 (39.13)	239 (37.00)	341 (39.98)	115 (41.52)	>0.05
Insurance					
Medicaid	1742 (98.09)	642 (99.38)	853 (100)	247 (89.17)	
Other	50 (2.85)	4 (0.62)	0	30 (9.39)	<0.0001

Data are mean ± SD or *n* (%). *p*-value reflects *p* for trend by ANOVA or *p*-value by chi-square test. ^a^ *p* < 0.0001 vs. Caucasian. ^b^ *p* < 0.01 vs. Hispanic. ^c^ *p* < 0.001 vs. Caucasian. ^d^ *p* < 0.05 vs. Caucasian.

**Table 2:** Adverse pregnancy outcomes by ethnicity.

Characteristics	All Participants	African American	Hispanic	Caucasian	* p * -Value
*n*	1776	646	853	277	
Gestational age at delivery (week)	38.65 ± 2.25	38.55 ± 2.40	38.69 ± 2.19	38.75 ± 2.07	>0.05
Infant birth weight (g)	3209 ± 582	3128 ± 592 ^a,b^	3241 ± 568	3301 ± 577	<0.0001
Preterm delivery (<37 weeks)	203 (11.43)	84 (13.00)	87 (10.20)	32 (11.55)	>0.05
Early preterm delivery (<34 weeks)	61 (3.43)	26 (4.02)	24 (2.81)	11 (3.97)	>0.05
Late preterm delivery (34–<37 weeks)	142 (8.00)	58 (8.98)	63 (7.39)	21 (7.58)	>0.05
Preeclampsia	158 (8.90)	77(11.92)	56 (6.57)	25 (9.03)	<0.01
Gestational diabetes mellitus (GDM)	77 (4.34)	13 (2.01)	45 (5.28)	19 (6.86)	<0.001

Data are mean ± SD or *n* (%). *p*-value reflects *p* for trend by ANOVA or *p*-value by chi-square test. ^a^ *p* < 0.0001 vs. Caucasian. ^b^ *p* < 0.01 vs. Hispanic.

**Table 3:** Differences in cytokine/adipokine concentrations by ethnicity.

Cytokine/Adipokine	African American	Hispanic	Caucasian	* p * for Trend
Entry (*n* = 1776)	646	853	277	
Adiponectin (µg/mL)	16.50 ± 0.35 ^a,b^	17.95± 0.35 ^c^	19.62 ± 0.55	<0.0001
Resistin (ng/mL)	50.13 ± 1.07 ^d^	45.08 ± 0.93 ^c^	48.88 ± 1.67	<0.01
GMCSF (pg/mL)	199.55 ± 10.87 ^e^	165.43 ± 9.47	195.10 ± 16.97	<0.05
IL10 (pg/mL)	12.66 ± 2.63	12.35 ± 2.29	11.92 ± 4.11	>0.05
IL8 (pg/mL)	41.46 ± 4.81 ^c^	34.95 ± 4.19 ^c^	23.64 ± 7.51	>0.05
IL6 (pg/mL)	4.55 ± 0.51	4.03 ± 0.45	3.33 ± 0.80	>0.05
TNFα (pg/mL)	11.11 ± 0.60 ^e^	10.38 ± 0.52	9.55 ± 0.93	>0.05

Data are mean ± SE. Means were adjusted for age, pre-pregnant BMI, parity, and cigarette smoking as covariates in by ANOVA. Log10 transformation was used for data analysis. ^a^ *p*< 0.01 vs. Caucasian. ^b^ *p* < 0.0001 vs. Hispanic. ^c^ *p* < 0.05 vs. Caucasian. ^d^ *p* < 0.01 vs. Hispanic. ^e^ *p* < 0.05 vs. Hispanic.

**Table 4:** Associations of decreased adiponectin level with preterm delivery by ethnicity ^a^.

Type of Preterm Delivery	Unadjusted *n* (%)	AOR (95% CI) ^b^	AOR (95% CI) ^c^
All participants (*n* = 1776)			
Early preterm	23 (37.7)	1.84 (1.07, 3.17)	1.82 (1.03, 3.21)
Late preterm	43 (30.28)	1.39 (0.94, 2.05)	1.37 (0.92, 2.03)
All preterm	66 (32.51)	1.46 (1.05, 2.04)	1.51 (1.09, 2.09)
Term Delivery	378 (24.03)	reference	reference
African American (*n* = 646)			
Early preterm	13 (50.00)	2.20 (0.93, 5.17)	2.04 (0.85, 4.88)
Late preterm	24 (41.38)	1.84 (1.02, 3.30)	1.89 (1.05, 3.42)
All preterm	37 (44.05)	1.80 (1.10, 2.94)	1.71 (1.06, 2.75)
Term Delivery	176 (31.32)	reference	reference
Hispanic (*n* = 853)			
Early preterm	6 (25.00)	1.11 (0.42, 2.98)	1.17 (0.44, 3.14)
Late preterm	15 (23.81)	1.02 (0.54, 1.93)	1.03 (0.54, 1.95)
All preterm	21 (24.14)	0.94 (0.55, 1.62)	0.84 (0.50, 1.42)
Term Delivery	164 (21.41)	reference	reference
Caucasian (*n* = 277)			
Early preterm	4 (36.36)	2.92 (0.76, 11.27)	3.13 (0.80,12.25)
Late preterm	4 (19.05)	1.09 (0.33, 3.60)	1.23 (0.37, 4.16)
All preterm	8 (25.00)	1.61 (0.64, 4.02)	1.79 (0.70, 4.55)
Term Delivery	38 (15.51)	reference	reference

Abbreviations: AOR, adjusted odds ratio; 95% CI, 95% confidence interval. The AORs and 95% CIs were computed by multiple polytomous logistic regression analyses. Early preterm: <34 weeks of gestation; late preterm: 34–<37 weeks of gestation; all preterm: <37 weeks of gestation. ^a^ Decreased adiponectin was defined as the lowest quartile <11.34 µg/mL vs. other quartiles pooled. Odds ratio indicated the risk associated with early or late preterm delivery in women who had low adiponectin levels. ^b^ Models were adjusted for pre-pregnancy BMI, maternal age, parity, and cigarette smoking. Ethnicity was adjusted for analysis in all participants only. ^c^ Models were fully adjusted with exclusion of patients who were complicated with preeclampsia.

**Table 5:** Association of cytokine and adiponectin levels with preeclampsia and gestational diabetes mellitus (GDM) in all participants when preterm delivery cases were excluded from analysis ^a^.

Cytokine/Adipokine	Outcome Variables	Unadjusted *n* (%)	AOR (95% Cl)
Adiponectin (<11.34 vs. ≥11.34 µg/mL)	Preeclampsia	44 (30.34)	1.45 (1.00, 2.14) ^b^
	GDM	32 (41.56)	1.72 (1.05, 2.84)
	Normal controls	323 (22.97)	reference
IL10 (≥11.29 vs. <11.29 pg/mL)	Preeclampsia	39 (26.90)	1.11 (0.76, 1.65)
	GDM	11 (14.29)	0.50 (0.26, 0.96)
	Normal controls	352 (25.04)	reference
TNF-α (≥9.97 vs. <9.97 pg/mL)	Preeclampsia	40 (27.59)	1.14 (0.78, 1.69)
	GDM	18 (23.38)	0.77 (0.44, 1.35)
	Normal controls	346 (24.61)	reference
IL6 (≥2.57 vs. <2.57)	Preeclampsia	30 (20.69)	0.81 (0.53, 1.24)
	GDM	19 (24.68)	0.96 (0.56, 1.64)
	Normal controls	351 (24.96)	reference
IL8 (≥13.26 vs. <13.26)	Preeclampsia	32 (22.07)	0.85 (0.56, 1.29)
	GDM	23 (29.87)	1.07 (0.63, 1.82)
	Normal controls	351 (24.96)	reference
Resistin (≥59.77 vs. <59.77)	Preeclampsia	34 (23.45)	0.93 (0.62, 1.40)
	GDM	18 (23.38)	0.89 (0.52, 1.55)
	Normal controls	352 (25.04)	reference
GMCSF (≥212.79 vs. <212.79)	Preeclampsia	38 (26.21)	1.00 (0.68, 1.49)
	GDM	14 (18.80)	0.70 (0.39, 1.29)
	Normal controls	355 (25.25)	reference

For adiponectin, the quartile of interest was the lowest quartile vs. other quartiles pooled; for the other cytokines, the quartile of interest was the highest quartile vs. other quartiles pooled. AOR and 95% CI were computed by multiple polytomous logistic regression analysis. The odds ratio indicated that the risk associated with preeclampsia or GDM in women who had exposure in low adiponectin levels or had increased levels in other cytokines. ^a^ Models were adjusted for maternal age, parity, pre-pregnancy BMI, and cigarette smoking with exclusion of preterm delivery patients. ^b^ *p* for trend <0.05.

## Data Availability

Data presented in this study are available on request from the corresponding author.

## Supplementary Materials

The following supporting information can be downloaded at: https://www.mdpi.com/article/10.3390/jcm11113213/s1, Table S1: Differences in cytokine/adipokine concentrations at entry with adverse pregnancy outcomes; Table S2: Elevated cytokine and decreased adiponectin levels with preeclampsia and gestational diabetes mellitus (GDM) by ethnicity.
