# Supplementary material for: Decreased Adiponectin Levels in Early Pregnancy Are Associated with High Risk of Prematurity for African American Women
Source: J Clin Med. 2022 Jun 4;11(11):3213. doi: 10.3390/jcm11113213 (PMC9181315; doi:10.3390/jcm11113213)
Supplement: Supplementary file 1 [file jcm-11-03213-s001.zip › jcm-1672035-supplementary.pdf]

Table S1. Differences in cytokine/adipokine concentrations at entry with adverse pregnancy outcomes

	**Preterm with Preeclampsia**	**Preeclampsia**	**Preterm Delivery**	**GDM**	**Normal Control**
**n**	**42**	**103**	**148**	**77**	**1,406**
Adiponectin (μg/mL)	16.01 ± 1.39 [a]	18.19 ± 0.89[a]	17.56 ± 0.74[a]	14.79 ± 1.04[b]	17.87 ± 0.24
Resistin (ng/mL)	50.00 ± 4.18	44.97 ± 2.67	48.47 ± 2.23	48.13 ± 3.12	47.48 ± 0.72
GMCSF (pg/mL)	158.79 ± 42.68	160.50 ± 27.27	174.11 ± 22.71	163.38 ± 31.79	186.71 ± 7.38
IL10 (pg/mL)	8.79 ± 10.25	14.14 ± 6.55	26.86 ± 5.46	6.86 ± 7.64	11.16 ± 1.77
IL8 (pg/mL)	23.67 ± 18.89	22.71 ± 12.07	29.07 ± 10.05	32.51 ± 14.07	37.70 ± 3.27
IL6 (pg/mL)	2.37 ± 2.01	3.99 ± 1.29	3.78 ± 1.07	4.10 ± 1.50	4.21 ± 0.35
TNFα (pg/mL)	9.77 ± 2.32	10.36 ± 1.48	9.72 ± 1.24	9.06 ± 1.73	10.71 ± 0.40

GDM, gestational diabetes mellitus.

Data are mean ± SE that were adjusted for maternal age, pre-pregnancy BMI, cigarette smoking and ethnicity. Log10 transformation was used for data analysis.

Forty two women with preterm delivery were also complicated with preeclampsia; thirteen women with GDM were also complicated with preterm delivery and preeclampsia.

a. $p < 0.05$ vs. GDM
b. $p < 0.001$ vs. normal controls

Table S2. Elevated cytokine and decreased adiponectin levels with preeclampsia and gestational diabetes mellitus (GDM) by ethnicity

Cytokine/adipokine	Outcome variables	Unadjusted n (%)	AOR (95% Cl) [a]
**African American**			
Adiponectin (< 11.34 vs. ≥11.34 µg/mL)	Preeclampsia	24 (32.43)	1.09 (0.64, 1.88)
	GDM	8 (61.54)	2.24 (0.66, 7.57)
	Normal controls	153 (30.48)	1.00
IL10 (≥11.29 vs. <11.29 pg/mL)	Preeclampsia	25 (33.78)	1.52 (0.90, 2.56)
	GDM	2 (15.38)	0.50 (0.11, 2.39)
	Normal controls	126 (25.10)	1.00
TNF-α (≥9.97 vs. <9.97 pg/mL)	Preeclampsia	23 (31.08)	1.60 (0.93, 2.74)
	GDM	4 (30.77)	1.22 (0.35, 4.21)
	Normal controls	111 (22.11)	1.00
**Hispanic**			
Adiponectin (<11.34 vs. ≥11.34 µg/mL)	Preeclampsia	13 (27.08)	1.46 (0.74, 2.90)
	GDM	18 (40.00)	1.86 (0.95, 3.62)
	Normal controls	141 (20.41)	1.00
IL10 (≥11.29vs. < 11.29 pg/mL)	Preeclampsia	10 (20.83)	0.83 (0.40, 1.71)
	GDM	5 (11.11)	**0.38 (0.14, 0.99)**
	Normal controls	165 (23.83)	1.00
TNF-α (≥9.97 vs. <9.97 pg/mL)	Preeclampsia	13 (27.08)	1.03 (0.53, 2.01)
	GDM	9 (20.00)	0.58 (0.27, 1.27)
	Normal controls	177 (25.62)	1.00
**Caucasians**			
Adiponectin (<11.34 vs. ≥11.34 µg/mL)	Preeclampsia	7 (30.43)	**2.97 (1.08, 8.17)**
	GDM	6 (31.58)	2.21 (0.70, 7.04)
	Normal controls	29 (13.62)[c]	1.00
IL10 (≥11.29 vs. <11.29 pg/mL)	Preeclampsia	4 (17.39)	0.63 (0.20, 1.97)
	GDM	4 (21.05)	0.71 (0.21, 2.40)
	Normal controls	61 (28.64)	1.00
TNF-α (≥9.97 vs. <9.97 pg/mL)	Preeclampsia	4 (17.39)	0.46 (0.15,1.47)
	GDM	5 (26.32)	0.65 (0.20, 2.12)
	Normal controls	58 (27.23)	1.00

Adiponectin was defined as the lowest quartile vs. other quartiles pooled; other cytokines were defined as the highest quartile vs. other quartiles pooled.

a. Models were adjusted for maternal age, parity, pre-pregnancy BMI and cigarette smoking with exclusion of preterm delivery patients.
